# Identifying determinants for promoting public engagement via Chinese E-government TikTok in public health emergencies

**DOI:** 10.1371/journal.pone.0325967

**Published:** 2025-06-18

**Authors:** Jingjing Guo, Julia Wirza Mohd Zawawi, Syafila Kamarudin

**Affiliations:** 1 Faculty of Modern Languages and Communication, Universiti Putra Malaysia, Serdang, Selangor, Malaysia; 2 Institute for Social Science Studies, Universiti Putra Malaysia, Serdang, Selangor, Malaysia; Bangkok Thonburi University: Bangkokthonburi University, THAILAND

## Abstract

**Background:**

Social media have become vital tools for governments during public health crises, enabling the timely dissemination of critical information, health guidance, and public engagement. In China, public engagement through social media, particularly TikTok, presents unique challenges during crises. This study investigates the factors that predict public engagement via the Chinese government’s TikTok during a public health emergency, focusing on the roles of information quality, source credibility, multiple cues, immediate feedback, and trust as a mediating variable.

**Methods:**

The study is grounded in the Elaboration Likelihood Model (ELM) and Media Richness Theory (MRT) to frame the relationships between these variables. Data were collected through an online questionnaire survey employing convenience sampling, involving 614 respondents aged 18–40 from Hebei Province, China. Descriptive statistical analysis was performed using SPSS, and Structural Equation Modeling (SEM) was employed to test the hypotheses.

**Results:**

The findings indicate that information quality and immediate feedback are significantly positively associated with public engagement via the Chinese government’s TikTok. However, source credibility and multiple cues did not have a positive impact on public engagement. Additionally, the study reveals that trust mediates the relationships between information quality, source credibility, and immediate feedback with public engagement.

**Conclusion:**

These results underscore the importance of trust in fostering public engagement and highlight the potential for enhancing government communication strategies on social media during crises. The study suggests that improving the quality of information and providing timely feedback can significantly increase public engagement through social media platforms like TikTok, particularly in the context of public health emergencies.

## 1. Introduction

Effective communication during public health crises is essential for governments to build trust, reduce uncertainty, and alleviate social anxiety. The massive influx of health information highlighted communication as a critical factor in saving lives [1]. For public health organizations and governments, social media platforms are indispensable tools for disseminating vital information, supporting evidence-based decision-making, and facilitating real-time interaction with the general population [2]. Engaging with the public through social media also enables governments to enhance public understanding of their policies and crisis management strategies [3].

Social media, with its capacity for immediacy, visual content, and interactive dialogue, has become increasingly important for public engagement in times of crisis [4]. In particular, TikTok has become a crucial platform for information sharing and communication between government organizations and the public [5]. As of April 2024, TikTok has achieved over 4.92 billion global downloads and hosts more than 1.582 billion active users, making it the fifth most popular social media platform worldwide. In China, TikTok (known as Douyin) has witnessed remarkable user growth. As of March 2025, its monthly active user base exceeded 1 billion. The platform’s core demographic remains concentrated among individuals aged 18–35, who account for approximately 60% of total users. On average, users spend more than 1.5 hours per day on the platform, with liking, commenting, and sharing being the most common forms of interaction. These patterns underscore the platform’s dominant presence in the daily digital routines of younger Chinese citizens.

In China, government agencies have increasingly adopted TikTok to engage with the public, publish statements, issue notifications, and address public concerns [2]. According to the China Internet Network Information Center (CNNIC), by the end of 2020, 26,100 official TikTok accounts had been established across all 32 provinces. Public health organizations, such as provincial health committees, have leveraged TikTok to disseminate health education and COVID-19 updates [6]. Furthermore, collaboration between TikTok and public health experts has led to the creation of a centralized information hub, offering users reliable and scientific COVID-19 guidance [2].

Despite these efforts, challenges remain in achieving meaningful two-way engagement through government TikTok. Prior studies have noted that government agencies often treat social media as a one-way communication channel, focusing on information dissemination rather than fostering dialogue [7,8]. Furthermore, issues such as restrictions on comment sections, concerns over privacy, limited transparency, and perceived risks regarding information credibility continue to limit public trust and engagement [9]. These challenges are further exacerbated in China’s tightly regulated media environment, where pandemic-related information is subject to strict control, reducing opportunities for public interaction and constructive dialogue [10]. These constraints collectively undermine public engagement online [11], emphasizing the need to develop strategies for enhancing digital engagement during crises.

While previous research has focused extensively on more traditional, text-based social media such as Twitter, Sina Weibo, and Facebook [10], fewer studies have examined emerging short-video social media platforms, particularly TikTok, in the context of public health emergencies [7]. Existing studies on government TikTok accounts during crises have primarily focused on content attributes, such as video length, titles, and dialogue loop [7]. While these technical factors provide valuable insights, they do not sufficiently explain the underlying mechanisms that foster sustained and meaningful public engagement, particularly during public health crises. As TikTok continues to evolve into an indispensable source of information and a critical communication channel for government agencies, further empirical research is needed to examine how this platform can effectively promote public engagement during crises [5].

Furthermore, a growing body of research has examined online public engagement, identifying key influencing factors such as content themes, content styles, and message framing [12,13]. Previous research has suggested that action-oriented messaging from government accounts leads to higher engagement levels, as evidenced by retweets and likes [14]. However, these studies often fail to capture the complex dynamics of public engagement with government communication during public health crises. Such emergencies significantly heighten the public’s need for timely, credible, and relevant information [7]. In this context, TikTok, with its rapid content updates and short, visually rich videos, provides governments with an effective platform for quickly capturing public attention and disseminating health information. However, the fast-paced flow of content can contribute to cognitive overload, leading users to rely on peripheral cues, such as source credibility, rather than engaging in thorough evaluation of each message [15]. Therefore, understanding how information attributes and media features shape public engagement on government-operated TikTok during public health crises is essential.

In addition, public trust is a vital factor influencing public engagement [16,17]. Khan et al. [18] demonstrated a strong relationship between trust levels and public intentions to access government e-services. Increased accessibility to information via government agencies’ social media platforms has also been identified as an effective means of building long-term trust among the public [16]. However, prior research has not provided a comprehensive framework for understanding how trust can be effectively cultivated. Moreover, scholars have called for further exploration of public trust behavior toward governments on social media platforms, particularly focusing on its mediating role in public engagement [19].

Therefore, based on this background, the following research questions are formulated to guide this study. RQ1. Which factors significantly influence public engagement via Chinese government’s TikTok during public health crises? RQ2. Does trust mediate the relationship between the predictors and public engagement via government’s TikTok?

## 2. Literature review

### 2.1. Public engagement via the Chinese government’s TikTok

The nature of public engagement has evolved significantly with the rise of social media, leading to the emergence of a new category of users known as digital citizens or e-citizens [20,21]. Public engagement refers to the process by which individuals actively engage in dialogue, decision-making, and information dissemination through online platforms [21–23]. In the context of public health crises, the proliferation of short-video platforms such as TikTok has introduced new forms of engagement that leverage immediacy, visual richness, and algorithm-driven content dissemination. These characteristics make TikTok particularly effective in capturing public attention and facilitating rapid information dissemination during crises [24]. Public engagement through government TikTok during crises extends beyond passive information reception to active engagement, including likes, shares, comments, content co-creation, and engagement in challenges or campaigns launched by official accounts [25–27].

As the demand for TikTok videos continues to rise among the Chinese public during public health crises, understanding the factors that influence public trust and engagement with government TikTok accounts becomes increasingly important [25–29]. Prior research has explored how the content type and format of TikTok videos produced by the Chinese government contribute to public engagement. For instance, official TikTok videos that incorporate subtitles, hashtags, Mandarin narration, a duration of less than 60 seconds, and original music have been found to enhance public sharing and engagement during COVID-19 [26,28]. Moreover, users tend to interact more frequently with videos presented in the type of cartoons, documentaries, and occupational health promotion content [28]. Similarly, Li et al. [26] concluded that official TikTok videos containing risk messages and references to preventive measures significantly enhance public engagement. Chen et al. [25] further argued that videos addressing government responses to crises and providing guidelines are more likely to attract public attention and generate higher engagement levels during COVID-19. In a more recent study, Zhang et al. [29] analyzed 354 TikTok videos from the National Health Commission of China (NHCC) and found that content with clear instructional messages significantly increased public engagement. Additionally, Che et al. [24] suggested that during the COVID-19 pandemic, the Chinese government adopted uncertainty-reducing communication strategies on the official TikTok account of the Chinese National Health Commission to help the public build a more comprehensive understanding of the crisis based on existing information.

Despite the growing prominence of government-operated TikTok accounts in public health communication, empirical research on their effectiveness in fostering public engagement remains limited. Few studies have investigated the underlying mechanisms that drive engagement. Given the interactive features of TikTok, it is crucial to examine how information quality, source credibility, multiple cues, immediate feedback, and trust shape public engagement. This study addresses this gap by analyzing the impact of these factors, offering insights into effective government communication strategies on TikTok during public health crises.

### 2.2. Research hypotheses and model

This study adopts an integrated conceptual framework grounded in the Elaboration Likelihood Model (ELM) and Media Richness Theory (MRT) to investigate the determinants of public engagement via Chinese government TikTok accounts during public health crises.

ELM explains how individuals process persuasive information through two routes: the central route, which requires cognitive effort and relies on message quality, and the peripheral route, which relies on cues such as source credibility or visual attractiveness [30]. In the context of government TikTok use during public health emergencies, ELM provides a useful framework for understanding how users evaluate health-related information.

MRT posits that richer communication channels, which are capable of handling multiple cues and enabling rapid feedback, are more effective in reducing uncertainty [31]. TikTok, with its multimedia capacity and real-time interaction features, offers a high level of media richness. Variables like multiple cues and immediate feedback are aligned with MRT, helping explain how TikTok’s media features support engagement during health crises.

ELM and MRT provide a theoretical foundation for examining how cognitive and media-related factors interact to shape public engagement on government TikTok during public health crises, offering a comprehensive understanding of engagement on short video platforms.

#### 2.2.1. Information quality.

ELM posits that information quality is a critical determinant of informational impact, particularly when there is a high likelihood of elaboration [30]. Information quality refers to the persuasive strength of the arguments presented in an informational communication [32], which in turn affects an individual’s ability to comprehend the content [2,32]. In the context of public engagement with government social media, information quality encompasses the evaluation of the timeliness, completeness, and accuracy of the information disseminated by government agencies [33].

The public is more likely to share and accept information that accurately and promptly reflects developments in a given situation within the vast pool of social media content [34]. Therefore, the government’s ability to rapidly distribute accurate and timely information plays a crucial role in reducing uncertainty and alleviating public concerns [35]. Furthermore, high-quality, persuasive information can mitigate negative perceptions surrounding crises such as COVID-19 and promote greater online engagement [36]. Dessart et al. (2017) [37] argued that high-quality information on social media helps individuals gain a deeper understanding and fosters positive attitudes toward engaging with online communities. Li et al. [2] further explored how information timeliness positively influenced public engagement during the COVID-19 crisis.

H1 Information quality has a positive relationship with public engagement via Chinese government TikTok.

#### 2.2.2. Source credibility.

According to ELM, the peripheral route requires less cognitive effort, with receptive behavior shaped by cues related to the target action, such as identifying with sources or relying on decision heuristics [38]. Source credibility refers to the extent to which an information source is perceived by the information recipient as competent, credible, and trustworthy [33]. During public health emergencies, the public is increasingly relying on online platforms to seek and share crisis-related information [13]. When social media content is perceived as coming from highly credible organizations or individuals, people are more inclined to recognize and affirm the value of the information [7,11].

Research indicates that individuals are more inclined to trust direct communication from authoritative sources [39]. Local governments are often considered the most critical source of information during disaster responses [25,40]. Source credibility is crucial to the public, as it reflects the trustworthiness of the person or organization behind the information [41].

Previous studies highlighted that credible sources exert a significant persuasive effect on public engagement [7,42]. Social media users are more likely to engage with information from sources they trust [42]. Shah and Wei [11] observed a positive relationship between source credibility and online public engagement based on a survey of 630 social network service (SNS) users. Similarly, Chen et al. [7] found that source credibility plays a crucial role in the dissemination of government information, as evidenced by their analysis of 413 Chinese government accounts on Sina Weibo.

H2 Source credibility has a positive relationship with public engagement via Chinese government TikTok.

#### 2.2.3. Multiple cues.

According to media richness theory, communication media having multiple cues possess high levels of information richness, thereby reducing equivocality and uncertainty [43,44]. Due to character limitations on social media, users often incorporate additional elements such as URLs, special tags or symbols, images, links, and videos to enhance their messages [45,46].

Visual media, such as photos and videos, are more effective than plain text in eliciting emotional responses from viewers, thereby increasing user engagement and attention [20,47]. Social media users can decipher posts with higher media richness at a lower cognitive cost [48], and information richness could supply additional information cues that improve individuals’ social engagement [2].

Some studies have suggested that media richness reduces likes and comments [25,49]. Khan et al. [4] analyzed 2,132 photos posted on the official Facebook page of the Malaysian Ministry of Health and confirmed that photos had no significant effect on public engagement (by comments, likes, and shares). However, other studies concluded that media richness was positively correlated with engagement [50]. Cao et al. [51] argued that high-quality visual content exhibits distinct attributes that significantly impact user engagement on social media. Similarly, Xiao and Blanco [52] concluded that posts with images received 89% more likes and 150% more retweets on Twitter.

The public may be more attentive to emergency information including images, links, and videos than to text-only content, especially during a crisis. Richer information can foster decision-making by reducing uncertainty [44]. For instance, Xu and Zhang [15] identified tweets related to the Malaysia Airlines flight tragedy, and found that the presence of a video significantly influenced the number of retweets.

H3 Multiple cues has a positive relationship with public engagement via Chinese government TikTok.

#### 2.2.4. Immediate feedback.

MRT suggests that the immediacy of feedback is the extent to which a medium enables users to exchange rapid and comprehensible feedback during communication [44]. Immediate feedback is a media capability that enables users to send and receive messages instantly [53]. It promotes real-time conversation and facilitates social interactions [54–56]. In this study, immediacy of feedback refers to the government’s ability to promptly respond to messages on social media, engaging in direct dialogue with the public [49].

Immediate response can ensure that communication remains current and updated, enhancing satisfaction with information and subsequently encouraging behavioral intent [57]. Government responses may provide a foundation for satisfying information needs and building government-citizen relationships [58]. Government responses on social media are typically more concentrated on the organization’s engagement with stakeholders via official social media accounts [59]. Openness to interactive feedback from the organization during a crisis is essential for improving public participation, lowering tensions, and boosting organizational support [60]. Additionally, it can help reduce the perceived severity of a crisis among the general public [61,62].

Previous research has shown that public engagement increases when governments are more responsive to their citizens, as people are more likely to believe that their comments and demands have a real impact on policy decisions [63]. Kim et al. [64] showed that health agencies’ interactivity online greatly influenced the level of public engagement with their COVID-19-related content on Twitter by analyzing 203 U.S. public health agencies’ Twitter activity. Similarly, Chen et al. [10] identified the Chinese Health Care Commission’s official Sina Weibo account and found that feedback had a positive effect on citizen involvement.

H4 Immediate feedback has a positive relationship with public engagement via Chinese government TikTok.

### 2.3. Trust as a mediator

Trust is widely recognized by scholars as a multifaceted, complex, and ambiguous concept [39]. Consequently, the notion of trust can hold multiple meanings. Trust in government refers to citizens’ perceptions and confidence in the organization’s ability to fulfill its responsibilities, make informed decisions, and serve the public interest [65]. Furthermore, Arshad and Khurram [17] defined public trust in government as an attitude shaped by individuals’ evaluations of expected impacts of social media utilization in governance.

ELM posits that high-quality arguments represent strong information reliability and persuasive effectiveness [66]. By sharing accurate and timely information on social media, the government enables citizens to better understand its efforts to address public needs, ultimately fostering greater trust in governmental institutions [67]. Moreover, during public health emergencies, individuals tend to have heightened information needs, which drive them to focus more on content-intensive central routes. This increased demand leads them to actively seek reliable information for fulfillment and to choose a central path for information processing [10]. Therefore, it is essential for governments to promptly distribute accurate information to the public during a crisis, as the timeliness of information plays a crucial role in how the public evaluates the credibility of its sources [67,68].

Source credibility can influence perceptions of information reliability, which in turn impacts the degree to which individuals trust information on social media [69]. Moreover, information shared by government agencies on social media regarding major decisions is perceived as a reliable source, thereby strengthening public trust [17]. Similarly, Nabi, Zohora, and Misbauddin [70] contended that by delivering comprehensive updates on COVID-19 via social media, the government has established itself as a key information source, thereby enhancing public trust and engagement.

Furthermore, the development of a “communicative relationship” through rich media characteristics, such as responsive feedback and reduced ambiguity, can enhance public trust [71]. The public has a more comprehensive and transparent understanding of government actions addressing their needs and concerns via social media, leading to an improvement in citizens’ trust in their government [17]. Therefore, several scholars claimed that governments should respond promptly to address the public’s stress, anxiety, behavioral, and emotional concerns in order to strengthen public trust [65,72].

H5 The relationship between information quality and public engagement is mediated by trust.
H6 The relationship between source credibility and public engagement is mediated by trust.
H7 The relationship between multiple cues and public engagement is mediated by trust.
H8 The relationship between immediate feedback and public engagement is mediated by trust.

**Fig 1:**
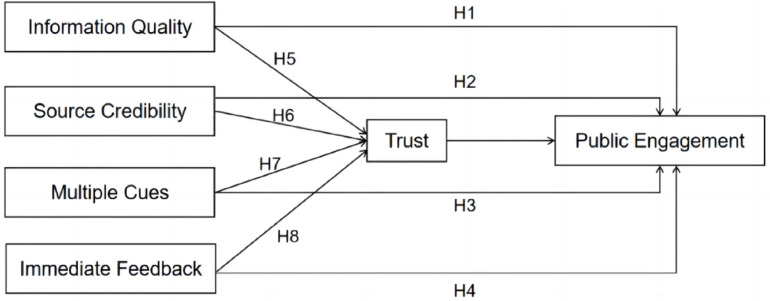
Conceptual framework.

Therefore, this study proposes the conceptual framework depicted in Fig 1.

## 3. Methodology

### 3.1. Measurement

To ensure the reliability and validity of the results, the questionnaire was developed based on well-established scales, with appropriate modifications to align with the research objectives and the specific context of TikTok. The main section of the questionnaire employed a five-point Likert scale, ranging from “strongly disagree” to “strongly agree”.

For information quality, items were adapted from Filieri et al. [73] and Lee et al. [12], with minor wording adjustments to reflect the TikTok environment, emphasizing objectivity, accuracy, and timeliness. Items measuring source credibility were adapted from Bhattacherjee and Sanford [32], focusing on the perceived trustworthiness and expertise of government-operated TikTok accounts. Multiple cues were adapted from Brunelle and Lapierre [74], capturing visual and symbolic elements relevant to TikTok’s multimedia format. The measurement of immediate feedback was based on Khan et al. [18], and adjusted to reflect TikTok’s real-time interactive features, such as comment responsiveness and update frequency. Trust was measured using adapted items from Khan et al. [18], revised to capture users’ confidence in the reliability of government information delivered through TikTok. Public engagement was measured by five items in reference to the scale of Shah and Lu [11]. The full list of measurement items, along with their sources, is presented in Table 1.

**Table 1:** The measurement items.

Items	Measures	Sources
**Information Quality**		
IQ1	The information posted by the organization on TikTok is timely.	Filieri et al (2015) Lee et al (2002)
IQ2	The information posted by the organization on TikTok is accurate.	
IQ3	This information posted by the organization on TikTok is objective and based on facts.	
IQ4	This information posted by the organization on TikTok is complete for my concerns.	
IQ5	This information posted by the organization on TikTok has sufficient breadth and depth.	
**Source Credibility**		
SC1	The organization on TikTok is credible.	Bhattacherjee & Sanford (2006)
SC2	The organization on TikTok is knowledgeable.	
SC3	The organization on TikTok is experienced.	
SC4	The organization on TikTok is reliable.	
SC5	This organization has the ability to understand and meet my needs.	
**Multiple Cues**		
MC1	While using TikTok, I can send/receive information through verbal and video.	Brunelle & Lapierre, 2008
MC2	While using TikTok, I can use a large pool of symbols to communicate (such as emotional icons/stickers/jpg/my pictures).	
MC3	Organization TikTok can transmite a variety of nonverbal cues (such as emotional tone/attitude).	
MC4	Organization TikTok allow people to use rich and varied language to communicate.	
MC5	Organization TikTok gives me a feel of face-to-face communication (such as live streaming).	
**Immediate Feedback**		
IF1	Organization response to my comments about COVID- 19 on TikTok.	Khan et al., 2021
IF2	It does not take long to express my reactions to others through organizationt TikTok.	
IF3	While using TikTok, I can know immediately what others think about my ideas.	
IF4	While using TikTok, I can let others know immediately what I think of their idea.	
**Trust**		
T1	I believe that organization are trustworthy.	Khan et al (2021)
T2	I expect that organizations on TikTok will not take advantage of me.	
T3	This organizations’ TikTok seems to be honest and truthful to me.	
T4	I believe that TikTok has enough safeguards to make me feel comfortable using it to engage in organization.	
**Public Engagement**		
PE1	I like, comment, and share information on organization TikTok to help people during COVID-19 crisis.	Shah & Lu (2022)
PE2	I follow official organizations TikTok.	
PE3	I engage in conversations on government TikTok (e.g., sending private messages) hoping to adopt suggestions or solve problems.	
PE4	I exchange information related to COVID-19 crisis on organization TikTok to seek or provide help to others in decision-making process.	
PE5	I upload information, videos, and other graphical contents related to COVID-19 crisis on TikTok	

### 3.2. Data collection

This study employed a cross-sectional online survey conducted between February 1 and March 30, 2024. The study was conducted in accordance with the Declaration of Helsinki and approved by the Ethics Committee of the University Putra Malaysia (JKEUPM) (ethical approval code: JKEUPM- 2023–1314) on 22 December 2023. The first page of the online questionnaire presented an informed consent form, which outlined the study’s background, methodology, objectives, and assurances of anonymity. Participants were required to click the “I agree” button after reviewing the study information, thereby providing their informed consent to participate.

To ensure content validity, two academic experts in media and communication reviewed all items for clarity, relevance, and theoretical alignment. Minor wording adjustments were made based on their feedback to improve contextual fit with the TikTok. Content validation typically involves two or more experts [75]. While three experts are often recommended to improve reliability and reduce potential bias, the use of two experts is considered acceptable when evaluations are consistent [76]. In this study, the two experts possessed relevant domain knowledge and provided consistent evaluations, supporting the adequacy of the content validation process. Future studies are advised to adopt a larger expert panel to strengthen methodological rigor.

Given the large population size of Hebei Province and the lack of a complete sampling frame for TikTok users who have accessed official government content, employing a probability sampling method was not feasible. Therefore, this study employed convenience sampling combined with screening questions to ensure that only participants who met specific criteria were included. The convenience sampling provides a cost-effective and time-efficient method for gathering data from a large number of respondents within a limited time. The sampling approach ensures a representative sample despite the use of an indefinite population. The inclusion criteria were: 1) Aged between 18 and 40 years. According to the All-China Youth Federation (ACYF), youth are defined as individuals within this age range. 2) Registered users of the TikTok. 3) Have accessed official information from the Chinese government on TikTok. 4) Reside in Hebei Province, China.

To ensure diversity in occupation, education level, and exposure to government TikTok content, respondents were drawn from hospitals, universities, companies, and communities across Hebei Province. These sectors were selected because they reflect different levels of varying levels of TikTok usage and engagement with government content, thereby contributing to a well-rounded sample. Specifically, 48.5% of the participants were students, recruited from universities such as Hebei Normal University, Hebei University of Engineering, and Handan University, located in different cities across the province. Additionally, 32.4% of the participants were full-time employees, working in companies situated in Shijiazhuang, Tangshan, Qinhuangdao, and Handan of Hebei. Participants were also selected from hospitals and community groups across various regions of Hebei, ensuring that both urban and rural areas were represented.

The sample size was calculated using the Taro Yamane formula (2003), n = N/ (1 + N·e^2^), with a confidence level of 95% and a margin of error of 0.05. According to the most recent data released by the National Bureau of Statistics, the total population of Hebei Province at the end of 2024 is 73.78 million. Of this, individuals aged 18–40 total 21.44 million, accounting for 29% of the overall population. Therefore, substituting this number into the formula yielded a recommended minimum sample size of approximately 400 participants. However, to ensure greater diversity and robustness, 630 individuals were invited to complete the questionnaire, and 614 responses were returned, resulting in a response rate of 97.4%. The relatively high response rate results in a larger data sample, narrower confidence intervals, and more robust statistical analysis [77]. Therefore, the 614 valid questionnaires will be used for data analysis.

### 3.3. Data analysis

The data analysis incorporated both descriptive and inferential statistics, with descriptive statistics serving as the initial step to provide a foundational understanding of the study’s participants and context [78]. Inferential statistics were applied to examine the relationships among variables and to test the proposed hypotheses.

Prior to conducting Confirmatory Factor Analysis (CFA) and Structural Equation Modeling (SEM), the normality of the data was assessed using skewness and kurtosis statistics. Based on the recommended thresholds (±2 for skewness and ±7 for kurtosis), all variables fell within the acceptable range, indicating that the data were approximately normally distributed and therefore suitable for CB-SEM (see Appendix A).

AMOS was employed to test the hypothesized relationships in this study. Initially, CFA was conducted for each component to evaluate the effectiveness of the indicators in measuring their respective constructs. Subsequently, SEM was conducted to assess the proposed model. Given that this study aims to empirically validate the relationships among variables based on theoretical assumptions, CB-SEM was deemed the most suitable SEM approach. AMOS, a widely used software for CB-SEM, was selected for its strong capabilities in confirmatory analysis, especially when working with large sample sizes (typically over 200 cases). Moreover, AMOS was preferred over Smart PLS and ADANCO for its robust framework in CB-SEM, which is designed for testing theory-based relationships and assessing model fit. In contrast, PLS and ADANCO are more suitable for variance-based SEM, which explores rather than confirms relationships [79]. Therefore, CB-SEM using AMOS was adopted, comprising two core components: the measurement model and the structural model.

The measurement model defines the relationship between observed variables and latent constructs, while the structural model assesses the hypothesized relationships among those constructs. Additionally, the reliability and validity of various constructs are assessed through multiple factors, including factor loadings, average variance extracted (AVE), Cronbach’s alpha, and composite reliability (CR). Subsequently, five goodness-of-fit indices were employed to evaluate the model fit, including the parsimonious fit index (ChiSq/df), incremental fit indices (CFI and TLI), and absolute fit indices (RMSEA and GFI). Furthermore, to examine the mediating effect of trust, a bootstrap method with 5,000 resamples was applied, using a 95% percentile confidence interval.

## 4. Results

### 4.1. Demographic profiles of the respondents

The results indicate that 55.4% of the respondents were female, while 44.6% were male. Regarding age, 48.5% of the respondents were between 18 and 23 years old. In terms of education, 66.3% held a Bachelor’s degree, and 17.7% had a Master’s degree. Additionally, 53.7% of the respondents were full-time students, while 6.4% were self-employed, and 7.3% were part-time employees. Table 2 presents a summary of the participants’ demographic characteristics.

**Table 2:** Distribution of youth by demographic characteristics (n = 614).

Profile	Frequency	Percentage(%)
Gender		
Male	378	61.6
Female	236	38.4
Age		
18-23	297	48.5
24-29	102	16.4
30-34	120	19.6
35-40	95	15.5
Education level		
Senior High School or Below	50	8.1
Diploma	49	8
Bachelor’s Degree	401	65.3
Master’s Degree	103	16.8
Doctor of Philosophy	11	1.8
Employment status		
Temporary/Part-time Employment	26	4.2
Full-time Employment	199	32.4
Self-employed	47	7.7
Full-time Student	298	48.5
Unemployed	44	7.2

### 4.2. Measurement model

CFA was performed separately for each latent variable as a preliminary step for the SEM analysis. Table 3 presents the results of the construct fit indices.

**Table 3:** CR, AVE and Cronbach’s α.

Variables	Items	Factor Loading	CR(>0.7)	AVE (>0.5)
**Information Quality**	IQ1	0.851	0.956	0.814
	IQ2	0.927		
	IQ3	0.920		
	IQ4	0.920		
	IQ5	0.891		
**Source Credibility**	SC1	0.885	0.955	0.811
	SC2	0.921		
	SC3	0.920		
	SC4	0.928		
	SC5	0.847		
**Multiple Cues**	MC1	0.886	0.943	0.768
	MC2	0.861		
	MC3	0.906		
	MC4	0.856		
	MC5	0.873		
**Immediate Feedback**	IF1	0.827	0.919	0.740
	IF2	0.877		
	IF3	0.866		
	IF4	0.870		
**Trust**	T1	0.832	0.935	0.742
	T2	0.887		
	T3	0.800		
	T4	0.889		
	T5	0.894		
**Public** **Engagement**	PE1	0.814	0.924	0.710
	PE2	0.854		
	PE3	0.822		
	PE4	0.866		
	PE5	0.856		

Convergent validity and discriminant validity are two key aspects of construct validity. Convergent validity assesses the extent to which a measure or variable correlates with other measures of the same construct in theoretically expected ways [80]. It can be evaluated using three criteria: factor loading, average variance extracted (AVE), and construct reliability (CR) [81].

The recommended standardized factor loading value for each factor is 0.5 or higher, with higher factor loadings indicating stronger convergent validity [82]. In this study, all observed items demonstrated strong standardized factor loadings ranging from 0.75 to 0.93, indicating good convergent validity. According to Hair et al. [81], if the modification index (MI) value exceeded 4.0, it indicated that the model could be improved. Therefore, based on both theoretical justification and MI values greater than 4.0, covariance paths were added between SC1 and SC4, T1 and T2, and PE3 and PE4. These modifications enhanced the model fit while maintaining the theoretical integrity.

Moreover, convergent validity was assessed through AVE, calculated using standardized factor loadings. All constructs demonstrated AVE values above the 0.50 threshold, indicating that they captured a substantial proportion of variance. Specifically, the AVE values ranged from 0.710 to 0.814, confirming the strong convergent validity of the constructs. The composite reliability (CR) of the measurement model assesses the precision of measuring the study’s latent constructs [80] and serves as an indicator of convergent validity. A CR value of 0.7 or higher is considered acceptable. In this study, all CR values met the recommended threshold, confirming the reliability of the constructs.

Discriminant validity assesses the degree to which constructs that are theoretically distinct exhibit low correlations with one another. It is evaluated by comparing the AVE values of each construct with the squared correlation coefficients between constructs [81]. In this approach, the square root of a construct’s AVE should be greater than the correlation coefficients between that construct and any other constructs. As shown in Table 4, all absolute correlation values were lower than the square root of the AVE. Therefore, no concerns regarding discriminant validity were identified, confirming that the measurement model meets the required discriminant validity criteria. Additionally, all constructs reported Cronbach’s alpha values between 0.957 and 0.974, indicating strong internal consistency and high reliability.

**Table 4:** Discriminant validity.

	IQ	SC	MC	IF	TRUST	PE
**Information Quality (IQ)**	**0.902**					
**Source Credibility (SC)**	0.702	**0.901**				
**Multiple Cues (MC)**	0.642	0.770	**0.876**			
**Immediate Feedback (IF)**	0.624	0.719	0.826	**0.860**		
**TRUST**	0.622	0.714	0.723	0.766	**0.861**	
**Public Engagement (PE)**	0.489	0.520	0.557	0.607	0.637	**0.842**

Note: The diagonal values (in bold) are the square root of the AVE of each construct.

### 4.3. Structural model

The structural model was assessed using multiple goodness-of-fit indices, with recommended thresholds requiring GFI, CFI, and TLI to exceed 0.9, RMSEA to be below 0.08 [83], and the chi-square to degrees of freedom ratio (χ^2^/df) to remain under 5. The results confirmed a good model fit, with GFI = 0.901, CFI = 0.975, TLI = 0.971, IFI = 0.974, RMSEA = 0.047, and χ^2^/df = 1.957.

**Fig 2:**
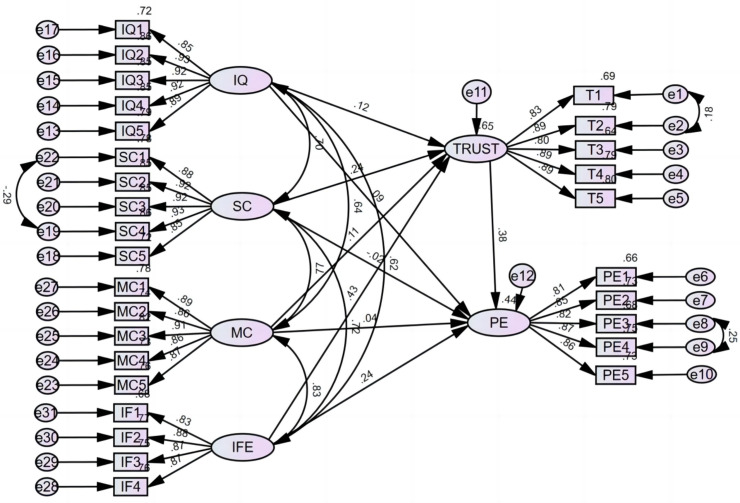
Result of the path analysis.

To evaluate the explanatory power of the model, the coefficient of determination (R^2^) values for the endogenous variables were assessed. This estimate reflects the variance in the results, which is explained by the predictor constructs. The R^2^ value represents the proportion of variance in the dependent variable that is explained by the independent variables. Higher R^2^ values indicate a stronger explanatory power of the model, while lower values suggest that additional factors may be influencing the dependent variable. In this context, values ranging from 0 to 0.10 indicate weak explanatory power, 0.11 to 0.30 represent modest explanatory power, 0.31 to 0.50 suggest moderate explanatory power, and values exceeding 0.50 signify strong explanatory power. As shown in Fig 2, the results of the structural equation modeling indicate that the R^2^ value of trust is 0.653, which means that information quality, source credibility, multiple cues, and immediate feedback collectively explain 65.3% of the variance in trust. This suggests that the predictors used in this study have a moderate-to-strong ability to account for variations in trust, supporting the validity of the theoretical framework. The R^2^ value for public engagement is 0.445, which falls within the weak-to-moderate range according to Hair et al. [81]. While this level is considered acceptable in behavioral and social science research, it also suggests that public engagement is influenced by additional factors not included in the current model.

### 4.4. Direct effects

**Fig 3:**
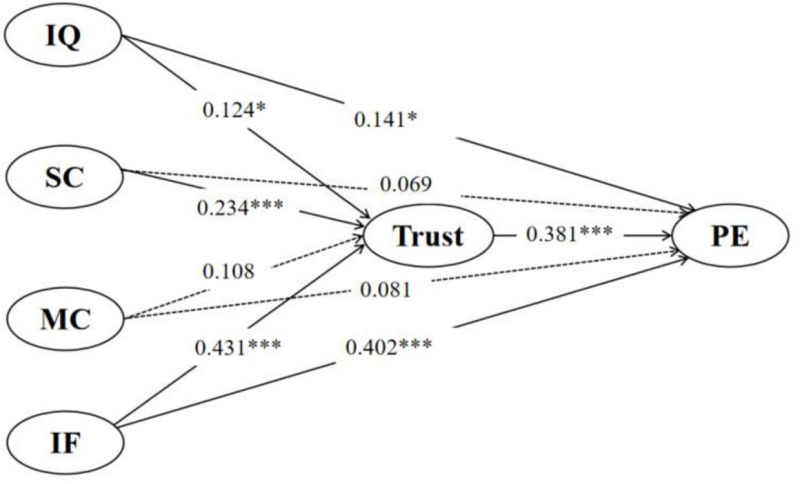
Analysis results. *p < 0.05. ***p < 0.001. IQ: information quality; SC: source credibility; MC: multiple clues; IF: immediate feedback; PE: public engagement.

The results of the analysis, as presented in Fig 3, indicate that information quality and immediate feedback have significant direct effects on public engagement via Chinese government TikTok, with standardized coefficients of 0.141 (p < 0.05) and 0.402 (p < 0.001), respectively (see Table 5). In contrast, the direct effects of source credibility and multiple cues on public engagement were found to be non-significant, with standardized coefficients of 0.069 and 0.081 (p > 0.05), respectively. Therefore, H1 and H4 are supported, while H2 and H3 are rejected.

**Table 5:** Hypothesis testing of the direct effect.

Hypothesized Relationship				Beta	S.E.	C.R.	P	Decision
H1	PE	<---	IQ	0.141	0.061	2.279	0.023^*^	Supported
H2	PE	<---	SC	0.069	0.083	0.898	0.369	Not supported
H3	PE	<---	MC	0.081	0.099	0.867	0.386	Not supported
H4	PE	<---	IF	0.402	0.095	4.527	p < 0.001^***^	Supported

* p < 0.05.

** p < 0.01.

*** p < 0.001.

### 4.5. Mediation effect

The results of the analysis, as presented in Fig 3, indicate that trust plays a significant mediating role in the relationships between information quality, source credibility, immediate feedback, and public engagement via government TikTok. Specifically, information quality (β = 0.124, p < 0.05), source credibility (β = 0.234, p < 0.001), and immediate feedback (β = 0.431, p < 0.001) all exhibit significant positive effects on trust, which in turn has a strong positive effect on public engagement (β = 0.381, p < 0.001). Furthermore, the indirect effects were found to be statistically significant within the 95% confidence interval (see Table 6). These findings suggest that trust partially mediates the effects of information quality, source credibility, and immediate feedback on public engagement. In contrast, multiple cues (MC) show neither a significant direct effect on public engagement (β = 0.081) nor a significant effect on trust (β = 0.108), indicating the absence of a mediating effect through trust. Therefore, H5, H6, and H8 are supported, while while H7 is not supported.

**Table 6:** Results of Bootstrap analysis.

Hypothesized Path	Beta	P	95%Bootstrap BC CI		Decision
			LB	UB	
H5: IQ→Trust→PE	0.057	0.005^**^	0.024	0.175	Supported
H6: SC→Trust→PE	0.108	0.002^**^	0.053	0.257	Supported
H7: MC→Trust→PE	0.05	0.240	−0.019	0.145	Not supported
H8: IF→Trust→PE	0.199	0.002^**^	0.132	0.347	Supported

* p < 0.05.

** p < 0.01.

*** p < 0.001.

## 5. Discussion

In response to the research questions (RQ1 and RQ2) posed earlier, this study examined the factors influencing public engagement via government TikTok during public health emergencies. The results found that information quality (H1) and immediate feedback (H4) were significant predictors of public engagement, addressing RQ1. Furthermore, trust plays a significant mediating role in the effects of information quality (H5), source credibility (H6), and immediate feedback (H8) on public engagement via government’s TikTok, which directly answered RQ2.

### 5.1. The influence of predictors on public engagement

The study’s findings reveal that information quality and immediate feedback significantly influence public engagement, supporting hypotheses H1 and H4, while source credibility and multiple cues did not have a significant effect, rejecting hypotheses H2 and H3.

Regarding Hypothesis 1, the findings reveal a significant positive influence of information quality on public engagement via the Chinese government’s TikTok during a public health emergency. This aligns with the central route of ELM, which posits that individuals motivated to process information are more influenced by high-quality content. In public health crises, accurate and timely government information fosters trust and encourages meaningful engagement, consistent with previous studies indicating that information quality is a key predictor of public engagement with government social media platforms [11,36]. Prior studies have demonstrated that high-quality content enhances users’ risk perception and encourages active engagement, particularly in online environments where information credibility and accuracy are crucial [11]. Furthermore, when governments disseminate comprehensive and up-to-date information, citizens are more likely to interact with, endorse, and share such content, ultimately leading to higher levels of public engagement [2].

Contrary to the expectations outlined in Hypothesis 2 and Hypothesis 3, this study found that source credibility and multiple cues have no significant positive impact on public engagement via the Chinese government’s TikTok during public health emergencies. This diverges from the assumptions of the ELM, which suggests that peripheral cues like source credibility and multimedia features influence low-effort processing. Contrary to prior research that emphasized the role of source credibility in shaping attitudes and behaviors, the findings suggest that during the COVID-19 crisis, citizens were more inclined to process information through the central route, focusing on the quality and accuracy of the content rather than the source or media richness [29]. This shift can be attributed to the public’s desire for accurate, consistent, and timely information to alleviate uncertainty and anxiety. In addition, despite the increased engagement driven by the government’s social media presence, the TikTok content still lacked comprehensive coverage of crucial information, such as COVID-19 prevention and transmission, which may have reduced the public’s trust and engagement.

Furthermore, prior research has emphasized that during crisis situations, the alignment between information demand and media supply is a critical determinant of communication effectiveness. Users tend to prioritize content clarity, relevance, and completeness over visual or stylistic complexity, as they seek actionable information that can reduce uncertainty and support informed decision-making [25,49]. The findings of this study further reinforce the notion that simple, accurate, and clear messaging is more effective times in fostering public engagement during public engagement of crisis.

Regarding Hypothesis 4, the findings reveal a significant positive influence of immediate feedback on public engagement via the Chinese government’s TikTok during a public health emergency. This finding is consistent with prior studies, which emphasize that openness to interactive feedback during crises fosters public trust, reduces uncertainty, and enhances engagement [59,61].

Moreover, timely and interactive responses from government agencies demonstrate their concern for public opinions, suggestions, and needs [84]. Such engagement can lead to positive emotional responses among citizens, including feelings of care, appreciation, and recognition. These emotions, in turn, cultivate a stronger sense of social belonging and identification with government organizations and online communities [49]. This sense of belonging has been shown to be a key driver of sustained public participation in digital spaces.

Therefore, the findings of this study reinforce the importance of active and responsive government communication on social media platforms, particularly during public health emergencies. Providing immediate feedback not only addresses public concerns in a timely manner but also helps alleviate public anxiety, strengthen perceived government transparency, and motivate continued public engagement.

### 5.2. Mediating role of trust

The findings from this study highlight that trust plays a significant mediating role in the effects of information quality, source credibility, and immediate feedback on public engagement via government’s TikTok. These results underscore the critical importance of trust in driving public engagement, especially during health crises. While the hypotheses related to the mediation effect of trust (H5, H6, and H8) were supported, the results did not support a mediating effect of trust in the path from multiple cues to public engagement (H7).

The importance of trust in crisis communication is consistent with prior research, which suggests that building and maintaining trust between the government and the public prior to a crisis is essential for ensuring effective communication and public compliance during emergencies [66]. In the context of this study, government credibility and public trust in information sources were crucial in encouraging engagement with TikTok content during a crisis. As the public generally relies on government-provided information during times of uncertainty, fostering this trust is a proactive measure that can significantly enhance engagement and cooperation during crises.

Furthermore, the study aligns with previous research emphasizing the critical role of source credibility in shaping trust in social media information [70]. The credibility of the information provided by government sources via TikTok directly influences public trust in the content, which in turn affects their willingness to engage with it. In this study, young people perceived government information about key decisions as credible, which led to increased trust and, ultimately, higher levels of engagement. This supports the notion that when governments use platforms like TikTok to disseminate official information, source credibility and the information quality play a crucial role in promoting engagement, particularly during public health crises.

TikTok, in particular, has emerged as a vital platform for disseminating information during the COVID-19 pandemic, further solidifying its value as a tool for crisis communication. The user-friendly interface of TikTok makes it an appealing platform for governments to connect with a younger, tech-savvy audience [29]. Facilitating prompt and two-way communication between the government and the public via TikTok fosters public trust by allowing the public to access relevant information and feedback provided by the agency, which creates a direct connection between the public and the government and promotes public trust. This is especially important in health crises, where the speed and accessibility of information can have a profound impact on public behavior.

### 5.3. Theoretical and practical implications

This study is grounded in an integrated theoretical framework that combines ELM, MRT, and trust to examine the online engagement behaviors of Chinese youth on TikTok. By synthesizing these established models, the research investigates how cognitive factors, media factors, and trust in information sources collectively shape public engagement in the digital realm. Notably, trust is identified as a critical mediating factor, highlighting its pivotal role in facilitating public participation during health crises, where the timely and reliable delivery of information is essential for alleviating public anxiety and fostering cooperation.

This theoretical integration and extension provide a significant contribution to the literature on government communication and public engagement in digital spaces. It bridges gaps between cognitive, media, and trust-based factors, offering a robust framework for understanding public behavior during crises. While ELM has traditionally been applied in marketing and consumer behavior research, this study extends its relevance to crisis communication, particularly during public health emergencies. This theoretical extension contributes to the literature by offering insights into how digital platforms like TikTok can be effectively utilized for governmental outreach and public engagement in times of crisis.

The findings of this study have several practical and policy implications. It is recommended that policymakers intensify efforts to build trust among youth and enhance online public engagement during crises. First, government agencies must ensure the delivery of accurate, consistent, and timely crisis-related information to foster public engagement on digital platforms. Establishing a robust content management system is essential for facilitating the prompt dissemination of crisis prevention and control measures, as well as for addressing misinformation and rumors that may arise during emergencies.

Second, governments should focus on establishing and maintaining the credibility of information sources. Although this study found no significant direct effect of source credibility on public engagement, prior research and our findings suggest that credibility indirectly influences engagement through trust. Consistency, transparency, and reliability in government communications are critical to strengthening public trust and fostering engagement.

In summary, to enhance public engagement via government TikTok accounts during public health emergencies, strategic planners should focus on strengthening the quality and accuracy of information, improving immediate feedback mechanisms, and building trust through sustained credibility and transparency. These efforts will ensure that government communication via social media platforms remains effective and responsive to public needs in times of crisis.

### 5.4. Limitations and future directions

This study has certain limitations. First, it examined the impact of information quality and source credibility as key factors influencing public trust and online engagement. Although the current model explains 44.5% of the variance in public engagement (R^2^ = 0.445), further studies could investigate additional central and peripheral factors, including perceived benefits, perceived risks, source attractiveness, and source expertise, which could offer deeper insights into user engagement behaviors, especially in the context of government communication on social media. Moreover, this study treated information quality as a unidimensional construct. However, information quality encompasses multiple dimensions, such as valence, informativeness, and helpfulness. Future research could investigate these dimensions individually to determine if that produces a different result on public engagement.

The interpretation of the findings may also be constrained by the research design used in this study. A potential concern is the accuracy and reliability of self-reported data, which may introduce limitations in data validity. Consequently, this may lead to reporting bias. Future researchers should consider conducting experimental studies to examine the causal effects of specific treatments on outcomes while controlling for potential confounding variables. Moreover, while quantitative research focuses on numerical data and statistical analysis, it may not fully capture the underlying motivations behind individual behaviors. Therefore, future studies are encouraged to incorporate qualitative approaches to gain deeper insights into participants’ behaviors, motivations, and perceptions, thereby enhancing the overall value of the research.

This study focused on Hebei Province to investigate youth engagement with government TikTok during a public health emergency. While the findings provide valuable insights, the regional scope may limit the generalizability of the results to other provinces or international contexts. Future research should extend the empirical analysis to multiple regions or conduct cross-cultural comparisons to validate the current findings and enhance external validity.

Finally, a key limitation of this study is its focus on young adults (18–40 years old) as the primary respondent group. Future research should consider expanding the age range and ensuring balanced representation across different age groups. This broader approach may bring more comprehensive and insightful findings.

## Appendix A

### Assessment of the Normality

**Table:** 

Variable	Minimum	Maximum	Skewness		Kurtosis	
	Statistic	Statistic	Statistic	Std. Error	Statistic	Std. Error
IQ1	1	5	-.819	.117	.345	.233
IQ2	1	5	-.496	.117	-.189	.233
IQ3	1	5	-.540	.117	-.111	.233
IQ4	1	5	-.510	.117	-.160	.233
IQ5	1	5	-.533	.117	-.192	.233
SC1	1	5	-.514	.117	.163	.233
SC2	1	5	-.470	.117	.091	.233
SC3	1	5	-.462	.117	.005	.233
SC4	1	5	-.405	.117	-.057	.233
SC5	1	5	-.543	.117	.164	.233
MC1	1	5	-.633	.117	.331	.233
MC2	1	5	-.554	.117	.028	.233
MC3	1	5	-.526	.117	.148	.233
MC4	1	5	-.442	.117	-.070	.233
MC5	1	5	-.550	.117	.157	.233
IF1	1	5	-.378	.117	-.129	.233
IF2	1	5	-.560	.117	.167	.233
IF3	1	5	-.484	.117	.056	.233
IF4	1	5	-.430	.117	-.022	.233
Trust1	1	5	-.494	.117	.130	.233
Trust2	1	5	-.492	.117	-.068	.233
Trust3	1	5	-.334	.117	-.049	.233
Trust4	1	5	-.337	.117	-.045	.233
PE1	1	5	-.466	.117	-.022	.233
PE2	1	5	-.489	.117	-.068	.233
PE3	1	5	-.387	.117	-.234	.233
PE4	1	5	-.428	.117	-.196	.233
PE5	1	5	-.381	.117	-.291	.233
